# The Role of the Apelin Receptor in the Pathophysiology of Pulmonary Arterial Hypertension

**DOI:** 10.3390/cells15050460

**Published:** 2026-03-04

**Authors:** Karla M. Rada, Alejandra M. Zúniga-Muñoz, Yamnia Q. Alvarez-Alvarez, Roxana Carbó, Horacio Osorio-Alonso, Cecilia Zazueta, Leonardo Del Valle-Mondragón, José L. Sánchez-Gloria, Gustavo Guevara-Balcázar, Ivan Rubio-Gayosso, Fausto Sánchez-Muñoz

**Affiliations:** 1 Sección de Estudios de Posgrado, Escuela Superior de Medicina, Instituto Politécnico Nacional, Mexico City 11340, Mexico; kradap2200@alumno.ipn.mx (K.M.R.); yalvareza2100@alumno.ipn.mx (Y.Q.A.-A.); gguevarab@ipn.mx (G.G.-B.); arubio@ipn.mx (I.R.-G.); 2 Departamento de Fisiología, Instituto Nacional de Cardiología Ignacio Chávez, Mexico City 14080, Mexico; 3 Departamento de Biomedicina Cardiovascular, Instituto Nacional de Cardiología Ignacio Chávez, Mexico City 14080, Mexico; alejandra.zuniga@cardiologia.org.mx (A.M.Z.-M.); roxana.carbo@cardiologia.org.mx (R.C.); ana.zazueta@cardiologia.org.mx (C.Z.); 4 Departamento de Fisiopatología Cardio-Renal, Instituto Nacional de Cardiología Ignacio Chávez, Mexico City 14080, Mexico; horacio.osorio@cardiologia.org.mx; 5 Departamento de Farmacología “Dr. Rafael Méndez Martínez”, Instituto Nacional de Cardiología Ignacio Chávez, Mexico City 14080, Mexico; leonardo.delvalle@cardiologia.org.mx; 6 Department of Internal Medicine, Division of Nephrology, Rush University Medical Center, Chicago, IL 60612, USA; jose_sanchez@rush.edu

**Keywords:** pulmonary arterial hypertension, APJ receptor, endothelial dysfunction

## Abstract

**Highlights:**

**What are the main findings?**

- Activation of the apelin receptor (APJ) is associated with improved endothelial homeostasis in pulmonary arterial hypertension.
- Activation of the apelin receptor (APJ) attenuates vascular remodeling in pulmonary arterial hypertension.

**What are the implications of the main findings?**

- APJ reduces vascular tone and emerges as a promising therapeutic target in pulmonary arterial hypertension.
- APJ reduces pulmonary vascular resistance and attenuates right ventricular hypertrophy in pulmonary arterial hypertension.

**Abstract:**

Pulmonary arterial hypertension (PAH) is a progressive disease characterized by endothelial dysfunction, vascular remodeling, and a sustained increase in pulmonary vascular resistance, causing cardiopulmonary damage. The apelin receptor (APJ), a member of the G protein-coupled receptor family, has emerged as an essential modulator of vascular homeostasis. Clinical and preclinical studies have demonstrated that its activation exerts beneficial effects on the progression of PAH. Its main actions include the restoration of endothelial function, reactivation of the BMPR2/SMAD axis, induction of nitric oxide-mediated vasodilation, inhibition of autophagy and the migration of the pulmonary artery smooth muscle cells (PASMCs). Furthermore, its expression and functionality are modulated by epitranscriptomic mechanisms, particularly by microRNAs involved in the post-transcriptional regulation of key genes for vascular homeostasis. These findings position the APJ as a relevant therapeutic target in PAH. However, the clinical application of its agonists still faces pharmacokinetic limitations that restrict their therapeutic use. Therefore, the aim of this review is to gather current information on APJ in the pathophysiology of PAH and focus attention on its potential as a therapeutic target.

## 1. Introduction

Pulmonary arterial hypertension (PAH) is a progressive cardiopulmonary disease. It is characterized by the remodeling of the pulmonary arteries, a sustained increase in the mean pulmonary arterial pressure (mPAP), and an increase in the pulmonary vascular resistance (PVR), leading to right ventricular heart failure [1,2]. PAH is a complex, multifactorial, and incompletely understood pathology. However, endothelial dysfunction, vascular smooth muscle hyperplasia, and chronic inflammation represent central characteristics in its development. These processes are mediated by integrated molecular and physiological pathways that include alterations in intracellular signaling pathways; imbalances in vasoactive mediators, particularly involving the prostacyclin, nitric oxide, and endothelin pathways; mutations in bone morphogenetic protein receptor type II (BMPR2); and epigenetic modifications, key components of the pathogenic model of PAH [3,4,5,6,7]. Despite significant advancements in understanding PAH pathogenesis, effective therapeutic strategies remain limited [8]. As a result, PAH remains a life-threatening disease. At present, the median survival is approximately 7 years overall [9]. Given this scenario, it is crucial to study more pathways or targets involved in the development of PAH.

In this context, a receptor called apelin receptor (APJ), a seven-transmembrane protein that belongs to the family of G-protein-coupled receptors (GPCRs), has gained significance due to its role in essential vascular processes. Although initially identified as an orphan receptor, it is now acknowledged for its pleiotropic effects, which include improving endothelial dysfunction, exhibiting anti-inflammatory properties, and regulating vasoactive substances in response to various ligands [10,11,12,13]. Previous studies have shown that activating this receptor provides protective effects in PAH. This is especially true as it contributes to the preservation of endothelial function, which is one of the earliest and most determining events in disease progression. Therefore, the aim of this review is to gather current information on APJ in the pathophysiology of PAH and focus attention on its potential as a therapeutic target.

## 2. APJ: Characteristics and Physiological Relevance

APJ was first identified in 1993 due to its structural similarity to the angiotensin II type 1 receptor (AT_1_R), but it does not functionally respond to this ligand, being classified as an orphan receptor [14,15]. It is highly conserved between species, with 90% sequence homology between humans and rodents. Its structure is characteristic of GPCRs, with seven transmembrane domains, an extracellular ligand-binding region, and intracellular G protein-coupled domains [14]. APJ belongs to the class A GPCR family, which is expressed in a variety of tissues, including the lungs, heart, kidneys, brain, adipose tissue, vascular endothelium, and smooth muscle, suggesting pleiotropic roles in cardiovascular, metabolic, renal, and neurological regulation [16]. Apelin was initially identified as its primary ligand, which is the reason for its name. However, other endogenous peptides, such as Elabela/Toddler, were subsequently discovered to activate the receptor [17]. Furthermore, APJ has been shown to form homo- and heterodimers with other GPCRs, allowing its activation even in the absence of traditional ligands [18,19]. This evidence suggests that APJ activity is dependent on the type of ligand, the cellular microenvironment, and the conformation adopted by the receptor.

APJ signaling is complex and not limited to a single intracellular pathway. Its activation can trigger pathways that regulate cell survival, energy metabolism, migration, contractility, and vascular tone [20,21,22]. This versatility is due to both its ability to couple with different G proteins (such as Gi/o or Gq) and to the activation of independent pathways, including those mediated by β-arrestin [23]. This ability defines the phenomenon of “biased agonism,” in which different ligands induce specific signaling profiles.

Diverse physiological and pathological conditions regulate APJ expression. Hypoxia induces a biphasic regulation mediated by Hypoxia-Inducible Factor 1-alpha (HIF-1α); other stimuli, such as angiotensin II (Ang II), alterations in the BMPR2 axis, hemodynamic overload, and oxidative stress, also modulate its expression [21,22,24].

Despite progress in the structural and functional characterization of APJ, its signaling mechanisms remain to be fully elucidated. The existence of multiple endogenous ligands, their dimerization capacity, and tissue variability in their effects hinder their pharmacodynamic characterization. However, this complexity represents an advantage from a therapeutic perspective, as it makes a promising pharmacological target in trying to study diseases such as PAH.

## 3. APJ and Endothelial Dysfunction: Pathophysiological Association in PAH

The pulmonary endothelium is a semipermeable barrier that lines capillaries and arterioles, playing a crucial role in regulating vascular tone, permeability, hemostasis, and the immune response. In PAH, its dysfunction represents a key pathological event. It is characterized by decreased endothelium-dependent vasodilation, increased oxidative stress, and activation of proinflammatory mechanisms—conditions that promote an abnormal proliferative and migratory cellular phenotype. This actively contributes to the initiation, progression, and maintenance of pulmonary vascular remodeling and the development of a progressive hemodynamic deterioration [4,6,25,26].

### 3.1. Alterations in Nitric Oxide Metabolism

Alterations in nitric oxide (NO) signaling are a central component of endothelial dysfunction in PAH. In this context, multiple experimental models and human studies have documented disruptions in NO production and bioavailability, which are closely associated with a proinflammatory, proliferative, and remodeling state of the pulmonary endothelium.

Due to the hypoxic microenvironment in patients with PAH, endothelial metabolic reprogramming has been described, characterized by increased glycolytic dependence, dysfunction of oxidative phosphorylation, and alterations in glutamine metabolism. These modifications not only reflect an altered adaptive phenotype but also a critical functional vulnerability, as the cells showed high sensitivity to inhibition of these pathways. The microvascular location of the affected cells reinforces the idea that endothelial dysfunction in PAH originates primarily in the pulmonary microvasculature [27].

In hypoxia-induced models, endothelial dysfunction is accompanied by the activation of mitophagy pathways and an increase in reactive oxygen species (ROS), as well as a significant decrease in NO production [28]. Additionally, it is also accompanied by alterations in intracellular calcium handling, loss of vascular relaxation capacity, and reduced acetylcholine-induced NO production, resulting from endothelial nitric oxide synthase (eNOS) dysfunction [29].

**Figure 1:**
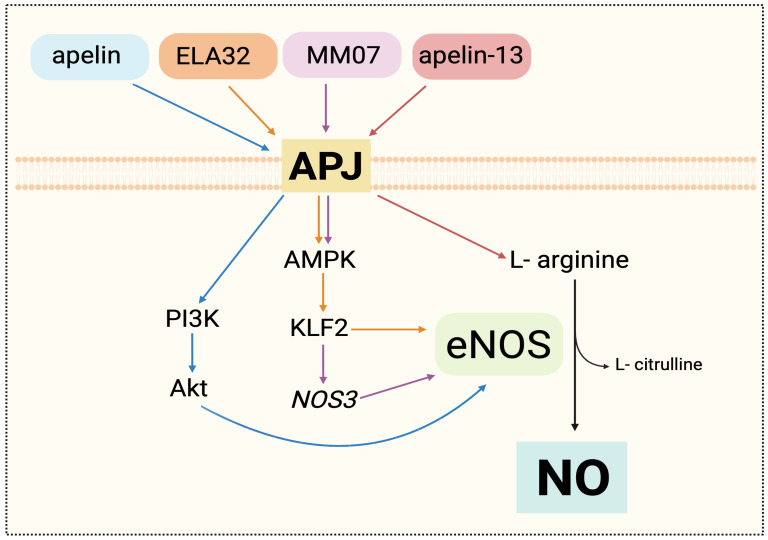
APJ-regulated nitric oxide synthesis. Phosphatidylinositol 3-kinase (PI3K); protein kinase B (Akt); AMP-activated protein kinase (AMPK); Krüppel-like factor 2 (KLF2); NOS3; endothelial nitric oxide synthase (eNOS); nitric oxide (NO). The binding of apelin, ELA32, MM07, and apelin-13 to the APJ receptor initiates the intracellular signaling pathways that promote NO synthesis. ELA32 and MM07 activate AMPK, encouraging the expression and activation of eNOS. Apelin stimulates the PI3K/Akt pathway, phosphorylating eNOS, and apelin-13 promotes the L-arginine uptake as a substrate for eNOS.

Under these pathological conditions, the APJ receptor emerges as a key regulator of NO metabolism, as shown in Figure 1. In the monocrotaline-induced PAH model, pharmacological activation of the receptor using the biased agonist MM07 restored AMP-activated protein kinase (AMPK) and eNOS phosphorylation, and increased NOS3 expression in human pulmonary arterial endothelial cells (PAECs), resulting in the recovery of the functional eNOS/NO axis (Figure 1) [30]. These molecular changes were associated with significant improvements in hemodynamic parameters such as right ventricular systolic blood pressure and ejection fraction [31]. In addition, in hypoxic models, the peptide apelin-13 enhanced L-arginine uptake, stimulated constitutive nitric oxide synthase (eNOS) activity, increasing NO concentrations at both tissue and plasma levels, and decreased the inducible nitric oxide synthase (iNOS) activity, which was linked to oxidative damage and inflammation [32]. APJ’s role in maintaining endothelial metabolism has also been seen in murine models of chronic hypoxia. An early overexpression of this receptor was observed in the first week of exposure, followed by a progressive loss of its expression. This reduction correlated with a significant decrease in serum nitrate levels, as well as lower expression and phosphorylation of eNOS. At the molecular level, APJ inhibition reduced the phosphorylation of AMPKα and acetyl-CoA carboxylase, in addition to suppressing the expression of Kruppel-like factor 2 (KLF2), a key transcriptional regulator of eNOS (Figure 1) [33]. In apelin-null models, the loss of KLF2 is more pronounced, suggesting the dependence on the apelin/APJ axis for the activation of this protective pathway. Notably, under physiological conditions, hypoxia stimulates KLF2 expression in PAEC, but this adaptive response is completely lost in the absence of apelinergic signaling [33].

Administration of an endogenous ligand of APJ, Apela (ELA32), by gene therapy, restored the expression of KLF2 and eNOS, indicating a reactivation of the KLF2/eNOS axis and an improvement in NO metabolism (Figure 1) [34]. At the structural level, prolonged deficiency of APJ receptor signaling in apelin-null models exposed to hypoxia was associated with severe microvascular rarefaction, evidenced by a reduction in the number of small-caliber arterioles (<75 μm) and the total number of vessels in histological sections. This vascular restructuring was a direct consequence of the suppression of the APJ/AMPK/KLF2/eNOS pathway, which promotes an inflammatory and remodeling environment, impairing NO bioavailability (Figure 1) [33]. Recently, Kim et al. [35] demonstrated that activation of the APJ receptor using extracellular vesicles functionalized with Apelin-13 and CARSKNKDC, a vascular homing peptide that selectively targets PAH-affected pulmonary endothelium, thereby indirectly enhancing APJ activation by increasing local apelin availability, promotes eNOS phosphorylation in PAECs from patients with PAH, indicating more efficient activation of the APJ–eNOS axis in the pathological endothelium. It is important to note that the functional activation of APJ was corroborated by the inhibition of forskolin-induced cyclic adenosine monophosphate (cAMP) production [35].

### 3.2. APJ-Mediated Inhibition of Autophagy and PASMC Migration

Apelin, through the APJ receptor, significantly reduced pulmonary artery smooth muscle cell (PASMC) proliferation without inducing apoptosis, suggesting a cytostatic rather than a cytotoxic effect in the hypoxia-induced PAH model. Furthermore, it inhibited cell migration and decreased autophagic vesicle formation, as evidenced by a reduction in microtubule-associated protein-1 light chain-3 (LC3), a protein used as a marker of autophagosome formation, punctate signaling, and monodansylcadaverine (MDC) positivity. This effect was associated with the activation of the PI3K/Akt/mTOR pathway, whose phosphorylation increased after apelin treatment. Pharmacological inhibition of this pathway with LY294002 reversed the anti-autophagic effects of apelin, restoring microtubule-associated protein-1 light chain-3-II (LC3-II) expression and suppressing Akt and mTOR activation. APJ specificity was confirmed by silencing it with a siRNA, which abolished PI3K/Akt/mTOR pathway initiation, restored autophagy, and eliminated apelin-induced antiproliferative and antimigratory effects. In APJ-deficient cells, apelin failed to reduce proliferation or LC3-II levels, confirming its strict dependency on the functional integrity of the APJ receptor [36].

### 3.3. Regulation of the Endothelial Phenotype

APJ receptor signaling regulates multiple aspects of the functional endothelial phenotype. In PAECs treated with MM07, an APJ agonist, a significant cell proliferation was observed, reaching levels comparable to those induced by recombinant human vascular endothelial growth factor (VEGF). In parallel, MM07 reduced TNF-α/cycloheximide-induced apoptosis, decreasing the proportion of Annexin+/PI− cells, without modifying basal apoptosis under conditions of serum and growth factor deprivation. This specific antiapoptotic action suggests a selective mechanism of protection against inflammatory damage [30]. Furthermore, gene therapy with AAV-ELA32, a vector that encodes Apela, an endogenous APJ ligand, successfully reversed the dysregulation observed in MCT-induced PAH models [34]. In this model, endogenous ELA32 and APJ receptor expression was restored, accompanied by marked inhibition of endothelial-to-mesenchymal transition (EndMT). This reversal was evidenced by reduced co-expression of von Willebrand Factor (vWF) and alpha-smooth muscle actin (α-SMA), as well as increased endothelial markers (vWF and CD34) and decreased mesenchymal markers (α-SMA and vimentin). Inhibition of EndMT therefore contributes to preserving the integrity of the endothelial barrier and limiting pulmonary arteriolar muscularization, one of the key events in the progression of PAH [34]. Additionally, in PAECs with suppressed APJ signaling, a marked increase in apoptosis was observed, accompanied by decreased cell proliferation and migration [34]. This functional impairment also compromised paracrine signaling between the endothelium and smooth muscle cells (SMCs), favoring a pro-remodeling environment. Restoration of the endogenous ligand reversed these effects, reducing apoptosis, restoring proliferative and migratory capacity, and attenuating vascular remodeling. Finally, in murine models with endothelial dysfunction induced by genetic deletion of an APJ transcriptional regulator, structural alterations characteristic of PAH were reproduced, including right ventricular hypertrophy (RVH), increased pulmonary systolic blood pressure, and muscularization of distal arterioles [37].

### 3.4. APJ/BMPR2 Bidirectional Axis

The functional loss of BMPR2 constitutes one of the best-characterized molecular mechanisms in the pathophysiology of PAH [38,39]. Its deficiency alters the balance between proliferation and apoptosis in endothelial and SMCs, favors the loss of microvasculature, promotes vascular remodeling, and contributes to the development of obliterating lesions [3,40,41]. Mutations in BMPR2 have been found in approximately 80% of familial cases of PAH and about 20% of idiopathic cases [42].

**Figure 2:**
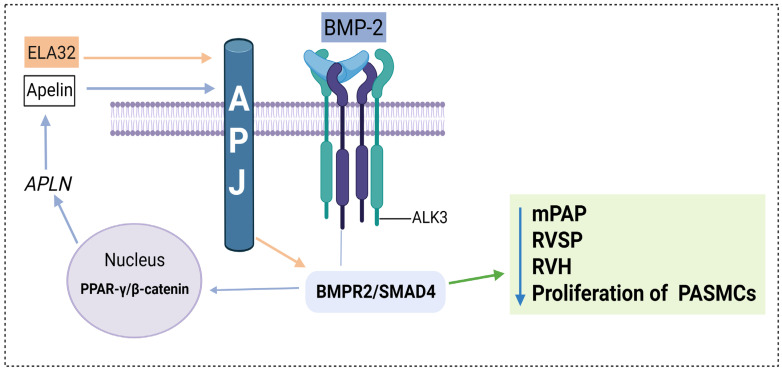
APJ/BMPR2 bidirectional regulation. ELA32 administration activates the apelin receptor (APJ), which stimulates bone morphogenetic protein type II (BMPR2) and SMAD4 signaling. BMP-2, through BMPR2 and ALK3, induces the nuclear formation of the peroxisome proliferator-activated receptor gamma (PPAR-γ) and β-catenin (PPAR-γ/β-catenin) transcriptional complex. This complex promotes the transcription of the APLN gene, which encodes apelin and activates APJ. Activation of APJ and BMPR2 is associated with reduced mean pulmonary arterial pressure (mPAP), right ventricular systolic blood pressure (RVSP), right ventricular hypertrophy (RVH), and pulmonary artery smooth muscle cell proliferation.

In this context, signaling between APJ and BMPR2 maintains a crucial interdependent relationship for pulmonary vascular integrity (Figure 2). In a monocrotaline-induced PAH model, administration of ELA32, an endogenous ligand of the APJ receptor, restored BMPR2/SMAD4 signaling in pulmonary arterioles and was associated with a reduced right ventricular systolic pressure (RVSP), attenuated RVH, decreased arteriolar muscularization, inhibition of the endothelial–mesenchymal transition, and preservation of the endothelial phenotype. Considering that this restoration is not observed in the absence of treatment, and ELA32 exerts its effects exclusively through APJ, this indicates that a reactivation of the BMPR2/SMAD4 axis occurs depending on the activation of APJ (Figure 2) [34]. On the other hand, APJ receptor activation requires the functional integrity of the BMPR2 axis. In human pulmonary endothelial cells, BMP-2 stimulation promoted functional activation of the BMPR2, leading to the nuclear formation of a transcriptional complex composed of peroxisome proliferator-activated gamma (PPARγ) and β-catenin. This complex directly regulates the transcription of the APLN gene, which encodes apelin, the endogenous ligand of the APJ receptor. BMPR2 inhibition significantly reduced apelin expression, limiting APJ activation and promoting SMC proliferation [37]. Furthermore, the PPARγ and β-catenin complex depends directly on the integrity of the BMPR2 axis, as its inhibition prevented the nuclear interaction between PPARγ and β-catenin. These findings establish a BMPR2-dependent transcriptional mechanism that regulates endothelial apelin availability and functionally conditions APJ receptor activation (Figure 2) [37]. These findings support the existence of a bidirectional functional axis: the APJ receptor stimulates BMPR2 expression, while BMPR2 receptor functionality is indispensable for BMP-2-induced signaling to promote APLN transcription, allowing APJ receptor activation [34,37].

Pulmonary vascular homeostasis does not depend exclusively on BMPR2-mediated signaling, but rather on the functional balance between this pathway and activin/transforming growth factor-beta (TGF-β) signaling, recognized as a central mechanism in the pathogenesis of PAH [43,44]. Activins, through their binding to activin type IIA (ACTR-IIA) and type IIB (ACTR-IIB) receptors, induce SMAD2/3 phosphorylation, activating responses associated with cell proliferation and vascular remodeling [9]. Pulmonary vascular integrity is critically regulated by the BMPR2/SMAD1/5/8 axis, associated with vasoprotective effects, and the activin/TGF-β/SMAD2/3 axis [45,46]. Alterations in this balance, characterized by reduced BMPR2 signaling and a predominance of activin/TGF-β signals, promote endothelial dysfunction and pulmonary vascular remodeling [39,43]. It should be noted that, although APJ signaling is functionally associated with the BMPR2/SMAD4 axis [34], current evidence does not support a direct interaction with activin signaling; therefore, its relevance should be understood in relation to BMPR2 regulation. In this context, sotatercept currently represents a clinically relevant example of a therapeutic strategy targeting dysregulated TGF-β/BMP superfamily signaling, acting as a ligand trap for activins and promoting the functional restoration of BMPR2 signaling [47,48].

## 4. Contribution of APJ to Physiopathogenesis of PAH

As described above, evidence indicates that modulation of the APJ receptor influences multiple molecular pathways that contribute to preventing or attenuating the hemodynamic, structural, and cellular alterations of PAH. This is, reduction in the expression of APJ and its ligands [33,37,49,50] is associated with disease severity. In contrast, short-term intravenous administration of (Pyr^1^) apelin-13 is associated with pulmonary hemodynamic improvement, evidenced by decreased pulmonary vascular resistance and increased stroke volume [51]. This effect was also observed in PAH patients treated with phosphodiesterase inhibitors (PDE5i), endothelin receptor antagonist (ERA), and prostacyclin, which was strengthened when those patients received PDE5i as concomitant therapy [51]. It was demonstrated that APJ contributes to the maladaptive right ventricular response to chronic pressure overload related to increased pulmonary vascular resistance, by using the agonist, MM07, that limits right ventricular dilation, partially normalizes end-diastolic and end-systolic volumes, and improves ejection fraction in the MCT-induced model [30]. Consistently, in the Su/Hx model, treatment with MM07 reversed the percentage of muscularized vessels to levels comparable with controls and significantly reduced the vascular smooth muscle score, an effect not observed with macitentan [31]. In both models, a decrease in RVSP, RVH, and vascular wall thickness were observed [30,31]. Consistent with these findings, Kim and colleagues demonstrated that APJ agonists, administered as Apelin-13 or delivered in extracellular vesicles (EV-CAR-Apelin and EV-Linker-Apelin), improved pulmonary arterial function, reflected in increased pulmonary acceleration time/pulmonary ejection time (PAT/PET) ratio, along with increased pulmonary vessel diameter, diminished right ventricular end-diastolic internal dimension, end-systolic internal dimensions, right atrial area, right ventricular end-systolic area and interventricular septum thickness [35]. In the same line, Apela administration reduced pulmonary arteriole muscularization and medial layer thickening, changes associated with decreased pulmonary arterial pressure in rats with PAH [34]. Chandra et al. demonstrated that apelin-deficient mice exhibited increased RVSP under chronic hypoxia compared with wild-type animals [33], besides significant pruning of the pulmonary microvasculature and increased muscularization of alveolar wall arteries with greater distal arterial loss [33]. Similarly, in TIE2CrePPARγfl/fl mice, reduced Apelin/APJ signaling was associated with PAH-related alterations including increased RVSP, RVH and pulmonary arterial muscularization [37]. Endogenous ligands suppressed all these changes, supporting a contributory role of APJ signaling in PAH pathophysiology. Finally, is worth mentioning that APJ stimulation reduced proliferation and induced apoptosis in PASMC, consistent with the attenuation of vascular remodeling observed in vivo [37].

## 5. Epitranscriptomic Mechanisms in PAH: The Role of microRNAs and APJ Receptor Signaling

**Figure 3:**
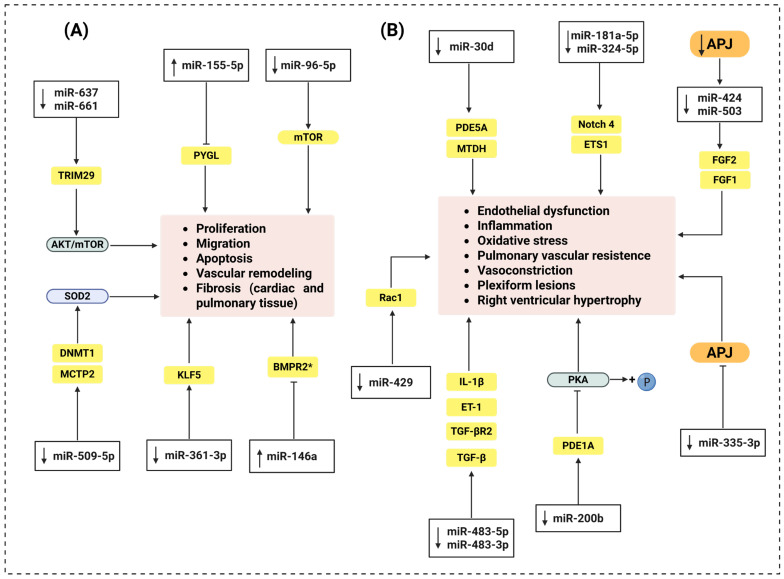
MicroRNAs’ role in pulmonary arterial hypertension (PAH). (**A**) Effects of miRNAs on regulation of pulmonary arterial smooth muscle cell (PASMC) proliferation. Decrease in miR-637 and miRNA-661 promote PASMC proliferation and migration by activating TRIM29, which activates the AKT/mTOR pathway. MiR-155-5p inhibits PYGL (phosphorylase glycogen liver form) PASMC proliferation. Decreases in miR-361-3p upregulate KLF5 (Krüppel-like factor 5) and decrease in miR-96-5p upregulates mTOR (mechanistic target of rapamycin), both favoring proliferation, migration, and vascular remodeling. Meanwhile, decreases in miR-509-5p promote MCTP and DNMT1, thereby increasing SOD2 expression and favoring proliferation. MiR-146a inhibits BMPR2 (bone morphogenetic protein receptor type II), promoting PASMC proliferation and reducing apoptosis. (**B**) Effects of miRNAs on endothelial dysfunction. Decrease in miR-483-3p and miR-483-5p enhances the expression of TGF-β (transforming growth factor-beta), TGFBR2 (transforming growth factor-β receptor type II), IL-1β (interleukin-1 beta), and ET-1 (endothelin-1), contributing to endothelial dysfunction. Decrease in miR-429-3p enhances Rac1 (Ras-related C3 botulinum toxin substrate 1), whereas decreased miR-200b upregulates PDE1A (phosphodiesterase 1A), inhibiting PKA (protein kinase A) phosphorylation, and decreased miR-30d upregulates PDE5A (phosphodiesterase 5A) and MTDH (metadherin), all favoring endothelial dysfunction, oxidative stress, vasoconstriction, and inflammation. Decreased miR-181a-5p and miR-324-5p upregulate ETS1 (ETS proto-oncogene 1 transcription factor) and Notch4 (notch homolog 4), exacerbating vascular remodeling and inflammation. Decreased miR-335-3p inhibits APJ, resulting in increased mPAP (mean pulmonary arterial pressure), RVH (right ventricular hypertrophy), RVSP (right ventricular systolic pressure) vascular remodeling, and plexiform lesions. APJ inhibition decreases miR-424 and miR-503, promoting FGF1 and FGF2 expression, which increases pulmonary vascular resistance, RVH, mPAP, and vascular remodeling. * Speculative target, + promotes phosphorylation, → activation; ⊣ inhibition. Color code: black, miRNAs; yellow, targets; blue, signaling components; orange, APJ (highlighted); light red boxes, outcomes.

MicroRNAs (miRNAs) are small noncoding RNAs, approximately 22 nucleotides in length, that have a critical regulatory post-transcriptional role in gene expression at multiple cellular processes; therefore, their dysregulation has been associated with numerous pathophysiological mechanisms, including PAH as summarized in Figure 3 and Table 1 [25,52,53,54].

Recently, miRNA studies have gained importance as regulators of molecular pathways in the progression of PAH, such as endothelial dysfunction, PASMC proliferation and migration, oxidative stress, inflammation, and vascular remodeling.

Jiang et al. [55] demonstrated that under hypoxic conditions, miR-637 and miR-661 levels are reduced in human PASMC, especially in those at higher clinical risk. These miRNAs downregulate the TRIM29 (Tripartite Motif Containing 29) gene, whose overexpression activates the AKT/mTOR signaling pathway, promoting cell proliferation and migration [55]. Hypoxia has also been observed to increase the expression of miR-155-5p, which is associated with increased expression of proangiogenic and hypoxic factors such as HIF-1α and VEGF. This activation promotes a proangiogenic, highly proliferative, and apoptosis-resistant cellular phenotype. Inhibition of this miRNA reverses these effects, slowing cell cycle progression [56]. In another study, Zhang et al. [57] determined that the circRNA circST6GAL1 is overexpressed in the blood of patients with PAH and the lung tissue of mice with monocrotaline-induced PAH. This circRNA acts as a “sponge” for miR-509-5p, which silences it. This silencing promotes the release of miR-509-5p, reversing hypoxia-induced proliferation and migration, and promotes apoptosis by the direct inhibition of MCTP2, a transmembrane protein involved in cell survival [57]. Similarly, miR-361-3p expression blocks PASMC proliferation, migration, and invasion by suppressing the translation of KLF5, a transcription factor linked to vascular senescence, inflammation, and cell growth [58]. On the other hand, miR-96-5p, overexpressed in PASMCs stimulated with PDGF-BB (platelet-derived growth factor-BB), a potent mitogenic growth factor involved in vascular remodeling, downregulates mTOR, a master regulator of cell metabolism and proliferation. It also activates the Uba1/Ube2n/Mdm2/ACE2 pathway, involved in regulating the cell cycle. The convergence of these mechanisms promotes excessive PASMC proliferation and thickening of the arterial media [59]. Regarding miR-509-5p, Wang et al. [60] demonstrated that restoring its expression in PASMCs significantly inhibits cell proliferation, migration, and promotes apoptosis. These effects are attributed to the inhibition of DNA methyltransferase 1 (DNMT1), which allows for the restoration of superoxide dismutase 2 (SOD2) expression, an essential antioxidant enzyme in the defense against oxidative stress [60]. MiRNAs also influence endothelial dysfunction and inflammatory responses associated with PAH. miR-483-3p and miR-483-5p have shown protective effects in monocrotaline- and Su/Hx-induced PAH models, reducing pulmonary arterial pressure and right ventricular hypertrophy. These miRNAs suppress the expression of TGF-β, its receptor transforming growth factor-β receptor type II (TGFBR2), β-catenin, IL-1β, and ET-1. Furthermore, a reduction in serum levels has been documented in patients with idiopathic PAH [61]. Furthermore, miR-200b and miR-30d exert beneficial effects by reversing the hemodynamic, structural, and functional alterations associated with PAH, mainly through modulation of the NO pathway. MiR-200b slows down phosphodiesterase 1A (PDE1A) expression, while the inhibition of miR-30d reduces sildenafil efficacy, a specific phosphodiesterase 5A (PDE5A) inhibitor [62,63]. On the other hand, Qi et al. demonstrated that the administration of exosomes enriched with miR-429-3p decreases oxidative stress, vascular remodeling, and inflammatory cell infiltration by inhibiting Rac1, a GTPase that regulates cell migration and the generation of ROS in PASMCs [64]. Sindi et al. [65] reported that the combined administration of miR-181a-5p and miR-324-5p decreases RVSP, RVH, and arterial muscularization. These miRNAs reduce the pulmonary expression of notch receptor 4 (Notch4), ETS proto-oncogene 1 (ETS1), α-SMA, and proliferating cell nuclear antigen (PCNA), as well as the activity of nuclear factor kappa-B (NF-κB), a key regulator in inflammatory signaling, hemodynamics, and vascular damage. Furthermore, they decreased VEGF-induced endothelial proliferation and blocked TNF-α and hypoxia-induced inflammatory activation in the Su/Hx model [65]. In Su/Hx and pulmonary artery banding (PAB) models, miR-146a inhibition attenuated RVH and decreased RVSP. In the monocrotaline-induced PAH model, its inhibition reduced the expression of BNP, a marker of cardiac dysfunction, as well as collagen type III alpha 1 chain (COL3A1). This gene encoding type III collagen contributed to an improvement in ventricular architecture [66].

**Table 1:** Role of miRNAs in PAH.

miRNAs	Expression in PAH	Target in Basal Conditions	Outcomes
miR-637 miR-661 [55]	↓ Expression	TRIM29	- Increases: PASMC proliferation and PASMC migration
miR-155-5p [56]	↑ Expression	PYGL	- Increases: cell proliferation, angiogenesis, and resistance to apoptosis
miR-509-5p [57,60]	↓ Expression	MCTP2 DNMT1	- Increases: PASMC proliferation and PASMC migration - Increases: oxidative stress
miR-361-3p [58]	↓ Expression	KLF5	- Increases: PASMC proliferation and PASMC migration
miR-96-5p [59]	↓ Expression	mTOR	- Increases: PASMC proliferation - Induces: medial layer thickening
miR-483-3p miR-483-5p [61]	↓ Expression	TGF-β TGFBR2 IL-1β ET-1	- Increases: mPAP, RVH, inflammation and fibrosis
miR-30d [62]	↓ Expression	PDE5A MTDH	- Increases: vascular remodeling, RVH and RVSP
miR-200b [63]	↓ Expression	PDE1A	- Increases: vascular remodeling, RVH and RVSP
miR-429-3p [64]	↓ Expression	Rac1	Increases: PASMC proliferation and PASMC migration
miR-181a-5p miR-324-5p [65]	↓ Expression	Notch4 ETS1	- Increases: RVSP, RVH, vascular remodeling and inflammation
miR-146a [66]	↑ Expression	BMPR2 *	- Increases: RVSP and RVH - Decreases: ventricular function
miR-424 miR-503 [50]	↓ Expression	FGF2 FGF1	- Increases: RVSP, RVH and vascular remodeling
miR-335-3p [67]	↑ Expression	APJ	- Increases: RVSP, RVH, mPAP vascular remodeling and PASMC proliferation

Abbreviations: TRIM29, tripartite motif containing 29; PYGL, phosphorylase glycogen liver form; MCTP2, multiple C2 and transmembrane domain-containing protein 2; DNMT1, DNA methyltransferase 1; KLF5, krüppel-like factor 5; mTOR, mechanistic target of rapamycin; TGF-β, transforming growth factor-beta; TGFBR2, transforming growth factor-β receptor type II; IL-1β, interleukin-1 beta; ET-1, endothelin-1; PDE5A, phosphodiesterase 5A; MTDH, metadherin; PDE1A, phosphodiesterase 1A; ETS1, ETS proto-oncogene 1 transcription factor; Notch4, notch homolog 4; BMPR2, bone morphogenetic protein type II; FGF2, fibroblast growth factor 2; FGFR1, fibroblast growth factor receptor 1; APJ, apelin receptor; PASMC, pulmonary artery smooth muscle cell; mPAP, mean pulmonary arterial pressure; RVH, right ventricular hypertrophy; RVSP, right ventricular systolic pressure; ↑ Expression, increased expression; ↓ Expression, decreased expression, * speculative target.

Although the role of miRNAs has been studied in multiple signaling pathways and target molecules widely implicated in the PAH pathophysiology (Figure 3), there are still unexplored molecular targets, specifically the APJ receptor. Kim et al. [50] identified an APLN/(miR-424, miR-503)–FGF2/FGFR1 regulatory axis that integrates key endothelial and paracrine functions for maintaining pulmonary vascular homeostasis. Their findings indicate that, under physiological conditions, APLN-mediated signaling induces the transcriptional expression of miR-424 and miR-503 in PAECs, which negatively regulate FGF2 and its receptor, FGFR1, thereby inhibiting endothelial proliferative activation [50]. They also observed a significant reduction in ligand presence without alterations in the APJ receptor, suggesting a functional disruption of the APJ/ligand axis. This dysregulation is associated with decreased levels of miR-424 and miR-503, which reduce the inhibition of FGF2 and FGFR1, triggering a sustained activation of pro-proliferative signals that promote endothelial hyperplasia and vascular remodeling (Figure 3 and Table 1). Furthermore, the authors identified a paracrine effect on PASMCs, which exacerbates proliferation and thickening of the arterial media [50]. In MCT and Su/Hx models, restoration of miR-424 and miR-503 expression reduced RVSP, RVH, and vascular remodeling, being associated with decreased FGF2 and FGFR1 levels in PAECs and lung tissue (Table 1) [50]. Additionally, another study conducted under chronic normobaric hypoxia (CNH) conditions showed that NF-κB signaling activates miR-335-3p transcription through binding of the p65 complex to its promoter [67]. This activation increases miR-335-3p expression in lung tissue, positively correlating with RVSP, RVH, and mPAP, which underlines its involvement in the clinical progression of PAH (Table 1). This miRNA acts as a direct post-transcriptional repressor of the APJ receptor, binding to its 3′-UTR region and reducing its protein expression in the lung [67]. APJ suppression compromises the protective signaling of the apelin/APJ axis, promoting hyperproliferation of PASMCs, inhibition of apoptosis, and vascular remodeling. Inhibition of miR-335-3p restores APJ receptor expression, decreases proliferation, and improves hemodynamic parameters. Furthermore, administration of the endogenous APJ ligand apelin-13 restored APJ function even in the presence of elevated miR-335-3p levels. Also, the treatment normalized the expression of proliferation (PCNA, α-SMA) and apoptosis (caspase 3, Bax) markers, which improved vascular architecture, reduced RVSP, and attenuated the progression of right ventricular hypertrophy. These findings highlight the relevance of the NF-κB/miR-335-3p/APJ axis as a central regulatory node connecting inflammation, structural remodeling, and hemodynamic dysfunction in PAH [67]. The evidence suggests that the APJ receptor not only participates in vascular regulation through its ligand apelin but is also under complex miRNA-mediated post-transcriptional control, which has direct functional implications in the pathophysiology of PAH.

### Pathophysiological Effects of miRNAs in PAH in the Context of APJ

In PAH, miRNA dysregulation is recognized as a key mechanism associated with endothelial dysfunction, abnormal proliferation, smooth muscle cell phenotypic alteration, and inflammatory activation [65,68,69]. Furthermore, miRNA modulation has shown therapeutic potential, including attenuation of vascular remodeling and hemodynamic improvement [69]. In human pulmonary artery smooth muscle cells (HPASMCs) exposed to hypoxia, overexpression of miR-637, miR-509-5p, miR-661, and miR-361-3p exerts antiproliferative and antimigratory effects and promotes apoptosis in pulmonary vascular cells [55,58,60]. Consistently, inhibition of miR-155-5p and miR-361-3p suppresses hypoxia-induced proliferation and migration, normalizes cell cycle distribution, and attenuates the expression of cell cycle regulatory proteins [56], changes associated with reduced pulmonary vascular remodeling characteristic of PAH. In monocrotaline models, restoration of miR-509-5p and miR-96-5p attenuates the increase in RVSP, reduces pulmonary vascular remodeling, and decreases RVH [57,59]. Specifically, miR-96-5p decreases the proportion of fully muscularized vessels and reduces fibrosis in pulmonary arteries and cardiac tissue [59]. A significant increase in miR-146a-5p and miR-155-5p will be observed in plasma extracellular vesicles and in the lung tissue of rats with PAH, associated with parameters indicative of greater severity, including HVR, elevated SPVR, increased mPAP, and increased vascular wall thickening [70]. Zhang et al. reported that decreased circulating is associated with greater disease severity in PAH patients [61]. Exogenous administration of miR-483 in Su/Hx and MCT models reduces mPAP, RVSP, and RVH and attenuates pulmonary arterial wall thickening, luminal occlusion, and pulmonary vascular remodeling [61]. In pulmonary endothelial cells exposed to hypoxia, miR-483 overexpression prevents hypoxia-induced increases in proliferation and migration [61]. Similarly, transgenic rats overexpressing miR-30d exhibit protection against pulmonary hypertension induced by MCT and Su/Hx [62]. MiR-30d overexpression reduces RVSP and mPAP, reverses RVH, and decreases distal pulmonary artery muscularization and medial wall thickening. Additionally, miR-30d exerts antiproliferative and antimigratory effects in hypoxic HPASMC [62]. On the other hand, Sánchez-Gloria et al. observed miR-21-5p upregulation associated with exacerbated pulmonary vascular remodeling, RVH, and increased pro-inflammatory cytokine levels in the MCT-induced PAH model [71]. In the MCT-induced PAH model, administration of extracellular vesicles enriched with miR-200b reduces proinflammatory mediators, including interleukin-6, tumor necrosis factor alpha, and interleukin-1 beta in the lung, and attenuates the increase in RVSP and medial remodeling of small pulmonary vessels [63]. Similarly, the transfer of miR-429-3p-enriched exosomes to hypoxically exposed PASMC attenuates proliferation and migration [64]. In mice with hypoxia-induced PAH, administration of miR-429-3p-enriched exosomes reduces pulmonary vascular remodeling, RVSP, and RVH [64]. Santos-Gomes et al. demonstrated that inhibition of miR-146a induces right atrial dilation, increased cardiomyocyte cross-sectional area (CSA), and medial wall thickening of pulmonary arteries in SuHx-exposed mice [66]. In the pulmonary artery banding (PAB) model, miR-146a inhibition reduces RVH and significantly decreases cardiomyocyte CSA [66]. Notably, specific microRNAs have been described whose modulation is directly associated with APJ regulation [50,67]. Reduced APLN/APJ signaling is associated with decreased miR-424 and miR-503, promoting vascular remodeling [50]. This alteration is accompanied by increased endothelial proliferation and enhanced PAEC capacity to induce pulmonary artery smooth muscle cell proliferation [50]. Consistently, in experimental models (MCT and SU5416/hypoxia), reduction in these miRNAs is associated with a characteristic PAH phenotype, including increased RVSP, RVH, microvascular muscularization, and vascular obliteration. These effects are abolished following restoration of APLN/APJ signaling and miR-424/miR-503 levels [50]. In the murine model of chronic normobaric hypoxia-induced PAH, increased miR-335-3p, which targets APJ, is associated with elevated RVSP, RVH, and pulmonary vascular remodeling. Pharmacological inhibition of miR-335-3p restores APJ signaling and attenuates RVSP, RVH, and vascular wall thickening [67]. Consistently, APJ activation with apelin-13 reproduces protective effects on pulmonary hemodynamics and vascular structure [67].

## 6. Clinical Potential and Current Limitations of APJ-Targeted Therapies in PAH

The above information suggests that the activation of the APJ receptor may represent a therapeutic target in PAH, as the activation by various types of agonists has been shown to induce favorable hemodynamic, structural, and functional effects in animal models and PAH patients. These findings support the characterization of APJ as a promising therapeutic target in this disease. All previous findings suggest that APJ manipulation may influence disease progression. In an interventional clinical trial involving patients with PAH, intravenous infusion of apelin-13 significantly decreased PVR, with a mean reduction of 14.9% compared to a placebo. Furthermore, a 19.5% increase in cardiac output and an 18.3% increase in cardiac index were observed, with no changes in heart rate, demonstrating a selective vasodilatory effect on the pulmonary bed and improved right ventricular function [51]. These effects were even more pronounced in patients receiving PDE5i, achieving a 28.4% reduction in PVR, suggesting a possible therapeutic synergy [51]. On the other hand, in a canine experimental model of acute pulmonary embolism, apelin infusion rapidly reduced mPAP mean and pulmonary capillary pressure, with an average decrease of 35%. A reduction in PVR accompanied these effects. In turn, an increase in stroke volume and cardiac index was observed, which will improve oxygen delivery (DO2) without compromising arterial oxygenation [49]. From an observational approach, in patients with PAH with chronic obstructive pulmonary disease (COPD) association, a significant reduction in the expression of the Apela/APJ system was identified in postmortem lung tissue. Compared with controls without PAH, lower Apela immunoreactivity was observed in endothelial, bronchial epithelial, and smooth muscle cells, accompanied by medial hypertrophy, muscularization of pulmonary arterioles, and intimal thickening. At the molecular level, the coexpression of vimentin and α-SMA, together with endothelial markers (CD34), suggests a hybrid phenotype involved in vascular remodeling processes [72]. Preclinical studies have explored the use of biased APJ agonists, designed to selectively modulate its activation by stabilizing specific conformations. Among them, MM07 has stood out for its greater bioavailability, plasma stability, and prolonged in vivo activity compared to apelin-13, showing comparable or superior effects in animal models of PAH [30,31]. Apelin-13 has a nanomolar affinity like that of apelin and is involved in processes such as vasculogenesis, cell migration, and cardiac output regulation during embryonic development. Furthermore, the identification of Toddler as an endogenous APJ ligand has expanded the functional understanding of the system [17].

Briefly, findings from experimental models, clinical trials, and human tissue studies suggest that the activation of APJ, regardless of the agonist used, can produce beneficial vascular and hemodynamic responses in the PAH context. These data reinforce the idea that APJ may represent a functionally essential therapeutic target, even without a meticulous characterization of the intracellular pathways involved. However, translating these findings into clinical application presents significant challenges. Pharmacological activation of the APJ is flourishing as an innovative strategy for the treatment of PAH. However, the agonists currently used in clinical and preclinical studies have limitations that hamper their large-scale therapeutic application. Among the most relevant challenges are the short plasma half-life of peptides such as apelin-13, its rapid degradation by endogenous peptidases, the need for intravenous administration, and its limited availability, which restricts its feasibility in outpatient settings [31,51]. Furthermore, the tissue distribution profile and low bioavailability of certain apelin or Apela analogs limit their sustained effectiveness. Although some compounds, such as MM07, have demonstrated greater stability and prolonged activity, the development of oral formulations, controlled-release parenteral formulations, or targeted delivery systems that allow for sustained therapeutic activation of APJ is still required [31].

These limitations highlight the need to develop new agonists that, in addition to maximizing the therapeutic benefits of the apelin/APJ axis, represent viable options in terms of accessibility and administration.

In this context, another possible option is to explore bioactive compounds present in commonly consumed foods that are easily metabolized, which represents a strategic alternative, particularly given their broad availability, low cost, tolerability, and potential to modulate the APJ receptor or its associated mechanisms functionally. One such example is (-)-epicatechin, which is recognized as one of the most abundant polyphenols in the human diet, and has been shown to exert vasodilatory, antioxidant, and antiproliferative effects [73,74]. Additionally, EPI has been reported to regulate both the expression and activity of the APJ receptor, suggesting possible therapeutic implications [75,76].

However, current treatment strategies for PAH are primarily centered on combination therapy [2]. While the restoration of APJ signaling and the inhibition of the activin pathway have independently shown promise [9,30,31,47,48,51], we hypothesize that a dual therapeutic approach may yield superior clinical benefits. Mechanistically, combining APJ agonism with activin signaling inhibition may provide synergy effects: the former reinforces essential vasoprotective mechanisms, while the latter suppresses maladaptive pro-remodeling pathways. Although the clinical application of such a combination remains unexplored, it represents a feasible strategy to explore in future studies. In conclusion, the paradigm of PAH treatment is shifting. Future strategies may require a comprehensive approach not only to suppress pro-remodeling pathways but also to restore and reinforce vasoprotective mechanisms essential for pulmonary vascular homeostasis.

## 7. Concluding Remarks

The reviewed evidence indicates that activation of APJ modulates key molecular and cellular pathways involved in the pathophysiology of PAH, particularly those associated with the preservation of endothelial function. Modulation of these pathways is associated with improvement in the pathophysiological features of the disease, including reductions in RVSP, mPAP, and RVH, as well as the prevention of medial wall thickening. Overall, these findings support APJ as a promising therapeutic target with the potential to prevent disease development and attenuate its progression.

## Data Availability

No new data were created or analyzed in this study.
